# Dendritic Mesoporous Organosilica Nanoparticles with Photosensitizers for Cell Imaging, siRNA Delivery and Protein Loading

**DOI:** 10.3390/molecules28145335

**Published:** 2023-07-11

**Authors:** Haneen Omar, Sara Jakimoska, Julia Guillot, Edreese Alsharaeh, Clarence Charnay, Frédérique Cunin, Aurélie Bessière, Jean-Olivier Durand, Laurence Raehm, Laure Lichon, Mélanie Onofre, Magali Gary-Bobo

**Affiliations:** 1 Chemistry Department, Collage of Science, Alfaisal University, Riyadh 11533, Saudi Arabia; ealsharaeh@alfaisal.edu,; 2 IBMM, University Montpellier, CNRS, ENSCM, 34193 Montpellier, France; sara_jakimoska@hotmail.com (S.J.); juliaguillot14@gmail.com (J.G.); laure.lichon@umontpellier.fr (L.L.); melanie.onofre@umontpellier.fr (M.O.); magali.gary-bobo@inserm.fr (M.G.-B.); 3 ICGM, University Montpellier, CNRS, ENSCM, 34193 Montpellier, France; clarence.charnay@umontpellier.fr (C.C.); frederique.cunin@enscm.fr (F.C.); aurelie.bessiere@umontpellier.fr (A.B.); jean-olivier.durand@umontpellier.fr (J.-O.D.); laurence.raehm@umontpellier.fr (L.R.)

**Keywords:** dendritic mesoporous organosilica nanoparticles, siRNA, FVIII factor

## Abstract

Dendritic mesoporous organosilica nanoparticles (DMON) are a new class of biodegradable nanoparticles suitable for biomolecule delivery. We studied the photochemical internalization (PCI) and photodynamic therapy (PDT) of DMON to investigate new ways for DMON to escape from the endosomes-lysosomes and deliver biomolecules into the cytoplasm of cells. We added photosensitizers in the framework of DMON and found that DMON were loaded with siRNA or FVIII factor protein. We made four formulations with four different photosensitizers. The photosensitizers allowed us to perform imaging of DMON in cancer cells, but the presence of the tetrasulfide bond in the framework of DMON quenched the formation of singlet oxygen. Fortunately, one formulation allowed us to efficiently deliver proapoptotic siRNA in MCF-7 cancer cells leading to 31% of cancer cell death, without irradiation. As for FVIII protein, it was loaded in two formulations with drug-loading capacities (DLC) up to 25%. In conclusion, DMON are versatile nanoparticles capable of loading siRNA and delivering it into cancer cells, and also loading FVIII protein with good DLC. Due to the presence of tetrasulfide, it was not possible to perform PDT or PCI.

## 1. Introduction

Mesoporous Organosilica Nanoparticles (MON), a new class of nanoparticles whose properties are different from those of well-known mesoporous silica nanoparticles (MSN) [1,2,3,4], have found applications in many different fields, and these nanomaterials have been comprehensively reviewed [5,6,7,8,9,10,11,12,13,14,15]. The applications mainly concern biology, with anticancer applications; however, environment decontamination [16] and catalysis [15] have also been studied, indicating a large field of applications for these nanoparticles. Indeed, the presence of the organic part leads to organic-inorganic hybrid materials, which are highly interesting due to the synergy between the organic and inorganic parts. These materials offer new properties and possibilities for applications. Hybrid organic-inorganic materials have many advantages over traditional materials. They can be tailored to have specific properties, such as high strength, flexibility, or conductivity. They can also be designed to be biocompatible, making them useful for biomedical applications. Overall, hybrid organic-inorganic materials have the potential to revolutionize many applications, including industrial ones, from electronics to medicine [17,18]. Combined with nanoscale technology, sophisticated core-shell systems have been designed for the sequential release of drugs [19]. In order to deliver larger biomolecules, large-pore MON have been designed. Two subclasses of MON are intricately structured MON (IMON) [20] and dendritic MON (DMON), which were very recently reviewed [21]. Yolk–shell or Janus-structured IMONs with separated compartments and with high surface area and pore volume rea useful for catalysis, drug delivery, and sensing applications. Deformable IMON allow for better penetration in cells and tumors. DMON have been mainly synthesized from bis(triethoxysilyl)ethane or bis(triethoxysilylpropyl)tetrasulfide. They possess radial channels in a three-dimensional network and a dendritic architecture. Their syntheses were carried out using three types of preparation methods [21]: aqueous phase system, the biphasic stratification system, and bicontinuous microemulsion system. We were interested in preparing DMON for the vectorization of nucleic acids such as siRNA [21] as they possess large pores suitable for this. Furthermore, mRNA vectorization [22] has also been studied and is very efficient. In the course of our studies of photodynamic therapy (PDT) and photochemical internalization (PCI) of siRNA [23], we were interested in the preparation of bis(triethoxysilylpropyl)tetrasulfide-based DMON for PDT and PCI of siRNA. PCI consists of using singlet oxygen to open the biological membranes—for example, the lysosomes—in order to deliver the biomolecule of interest in the cytoplasm of the cells [24]. For this, we covalently attached photosensitizers inside the framework of the DMON. We used three triethoxysilylated porphyrins and triethoxysilylated chlorin e6 as these photosensitizers proved efficient for PDT/PCI applications [25]. The method of synthesis we chose was the aqueous phase system, where no organic cosolvent was used. CTAB as a surfactant and sodium salicylate as a pore expending agent allowed to carry out the synthesis of DMON with radial mesopore structure. The photosensitizer was added at the beginning of the syntheses, with tetraethoxysilane and bis(triethoxysilylpropyl)tetrasulfide. We obtained nanoparticles with a 100 nm diameter, a large specific surface area, and a large pore size of 20 nm. The photosensitizers were detected with UV-Vis experiments. We then functionalized DMON with lysine amino acid in order to complex nucleic acids [26] thanks to multivalence offered by the grafting of lysine in the pores and at the surface of DMON. We also tested the encapsulation of the FVIII factor as a model protein to evaluate the potential of DMON in this field. 

## 2. Results and Discussion

First, the four commercially available photosensitizers PS1-4 were classically triethoxysilylated following the respective literature procedures [27,28,29,30].

**Figure 1:**
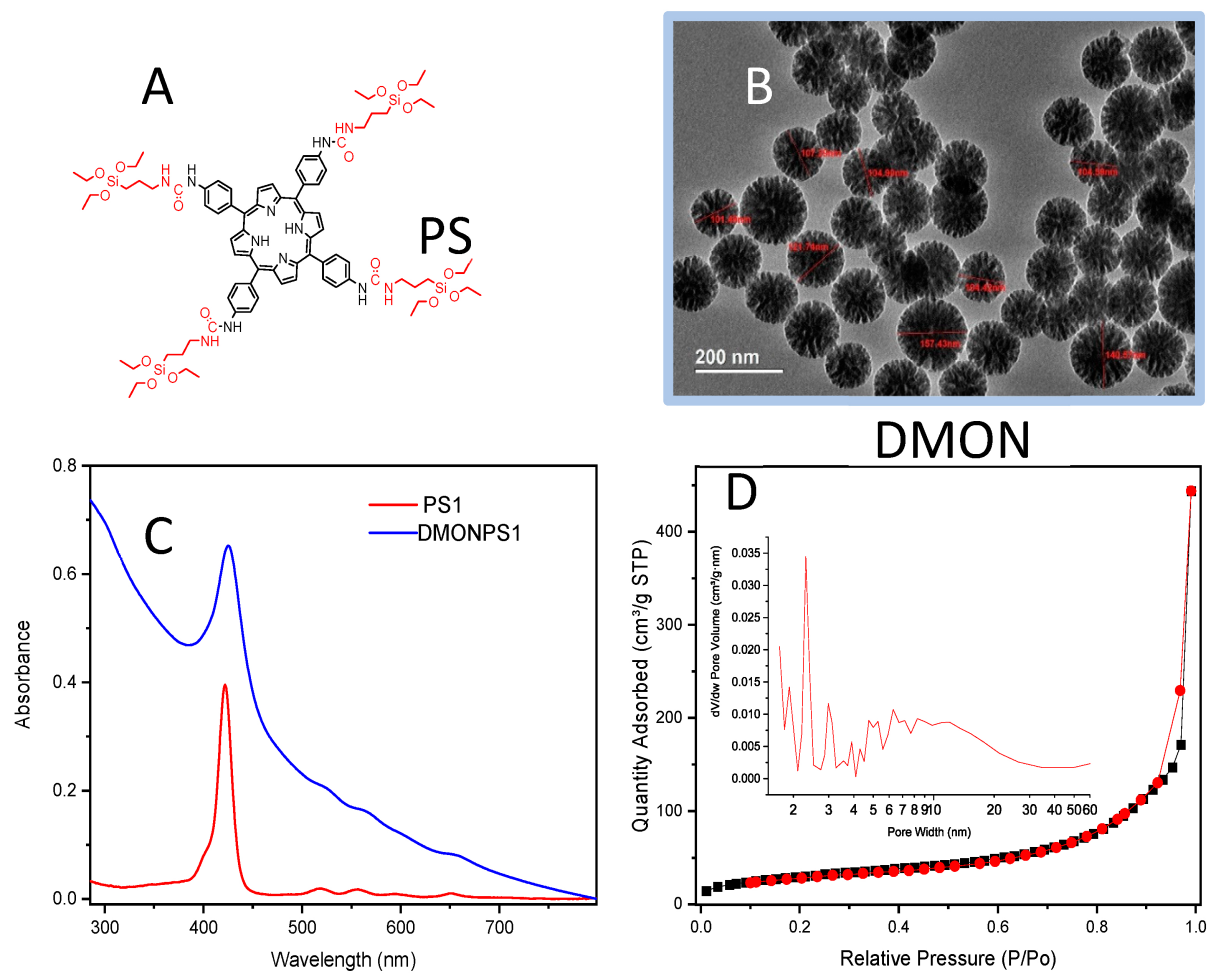
(**A**) The tetraaminophenylporphyrin with four triethoxysilyl groups was used for the preparation of DMONPS1. (**B**) DMONPS1 as shown by TEM, the dendritic structure is visible. (**C**) UV-Vis spectra in EtOH of the silylated photosensitizer (PS1) and the corresponding DMONPS1; the photosensitizer is encapsulated inside the framework of DMONPS1, and the Soret and four Q bands are visible. (**D**) Nitrogen adsorption–desorption at 77 K (BET). Red desorption, black adsorption. Insert BJH adsorption (dV/dw) pore volume.

**Figure 2:**
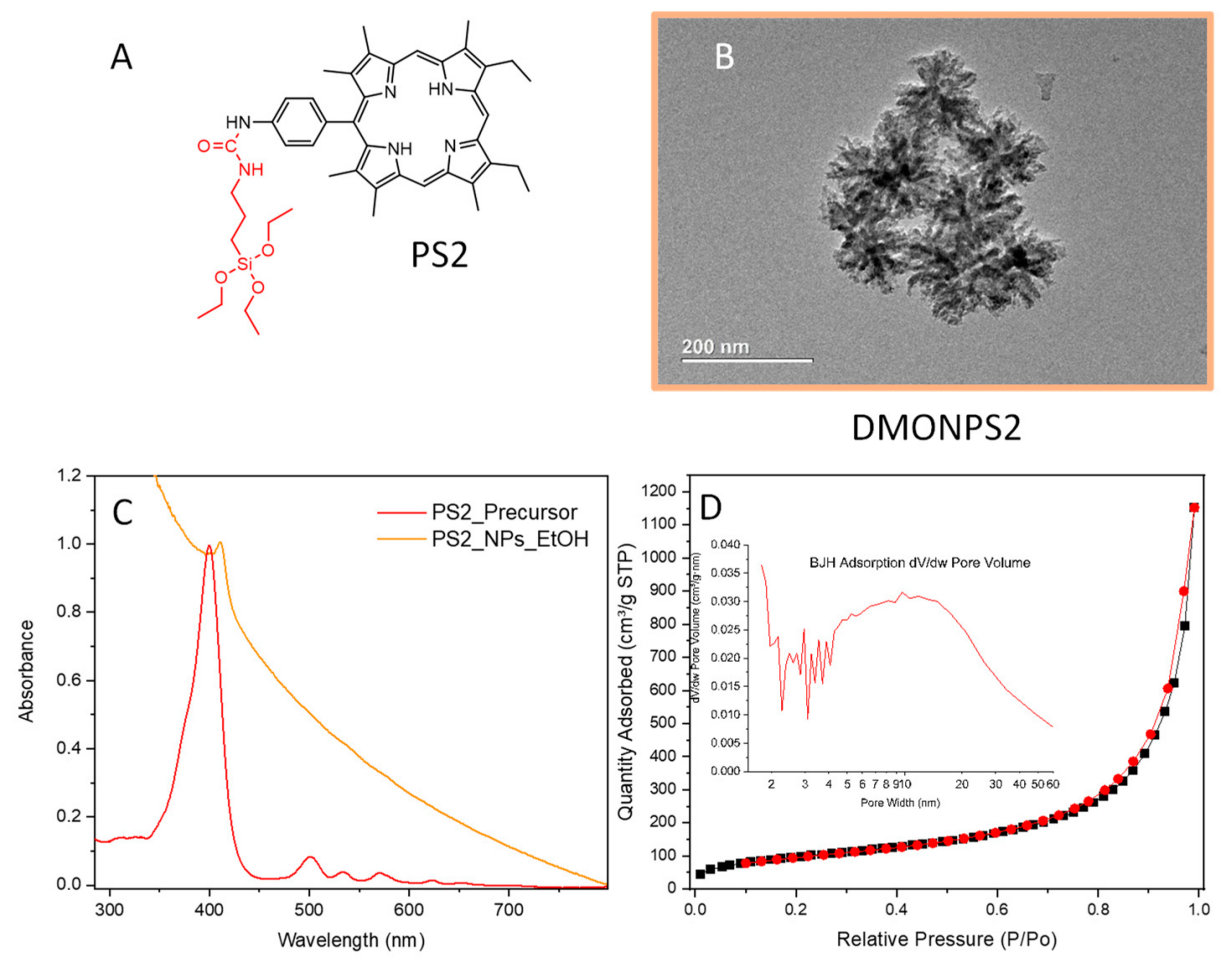
(**A**) The monosilylated aminophenylporphyrin was used for the preparation of DMONPS2. (**B**) DMONPS2 as shown by TEM: the dendritic structure is visible, and the nanoparticles seem aggregated. (**C**) UV-Vis spectra in EtOH of the silylated photosensitizer(PS2) and the corresponding DMONPS2; the photosensitizer is encapsulated inside the framework of DMONPS2, and the Soret band is visible. (**D**) Nitrogen adsorption–desorption at 77 K (BET). Red desorption, black adsorption. Insert BJH adsorption (dV/dw) pore volume.

**Figure 3:**
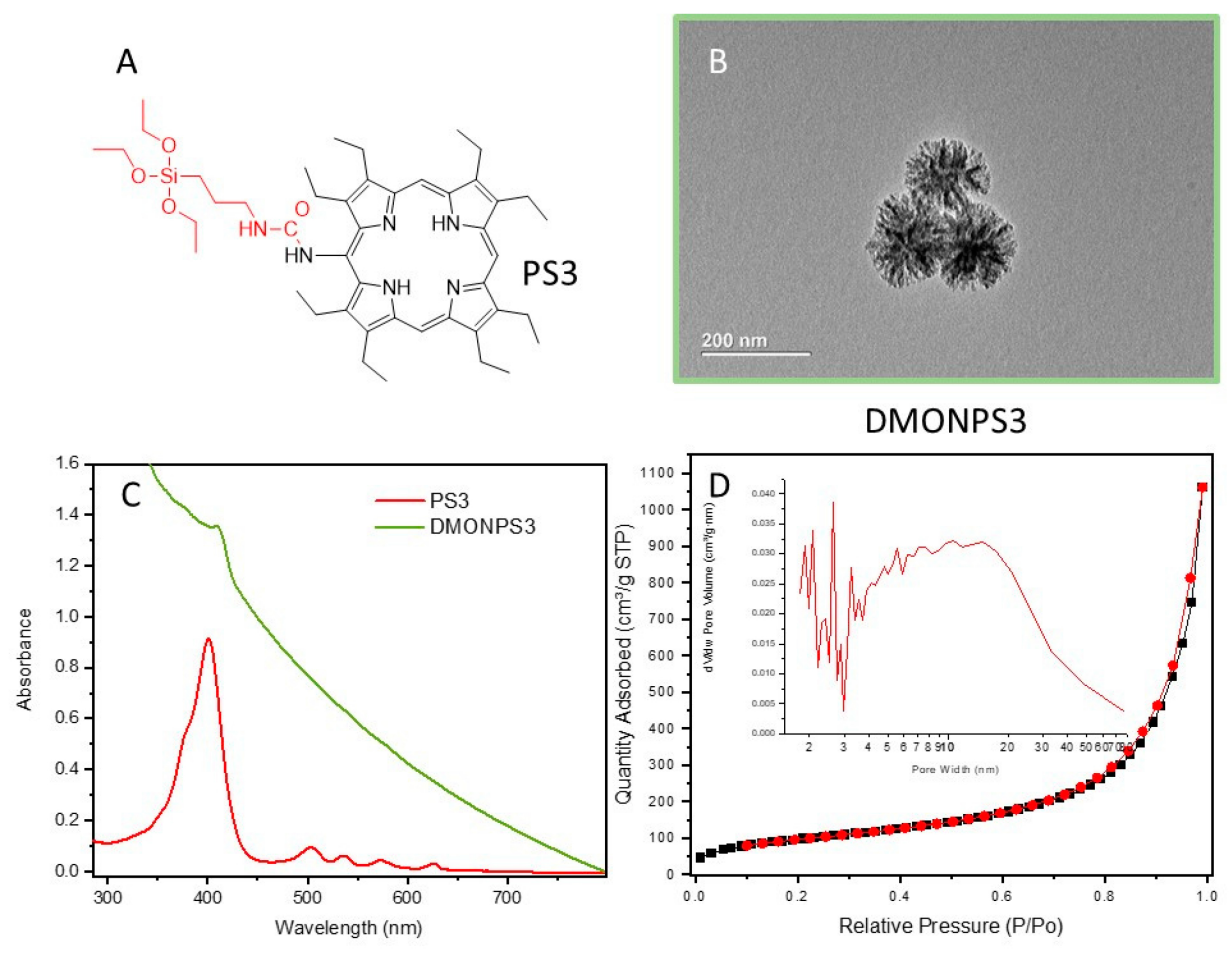
(**A**) The monosilylated aminoporphyrin used for the preparation of DMONPS3. (**B**) DMONP3 as shown by TEM: the dendritic structure is visible. (**C**) UV-Vis spectra in EtOH of the silylated photosensitizer(PS3) and the corresponding DMONPS3; the photosensitizer is encapsulated inside the framework of DMONPS3, and the Soret band is visible. (**D**) Nitrogen adsorption–desorption at 77 K (BET). Red desorption, black adsorption. Insert BJH adsorption (dV/dw) pore volume.

**Figure 4:**
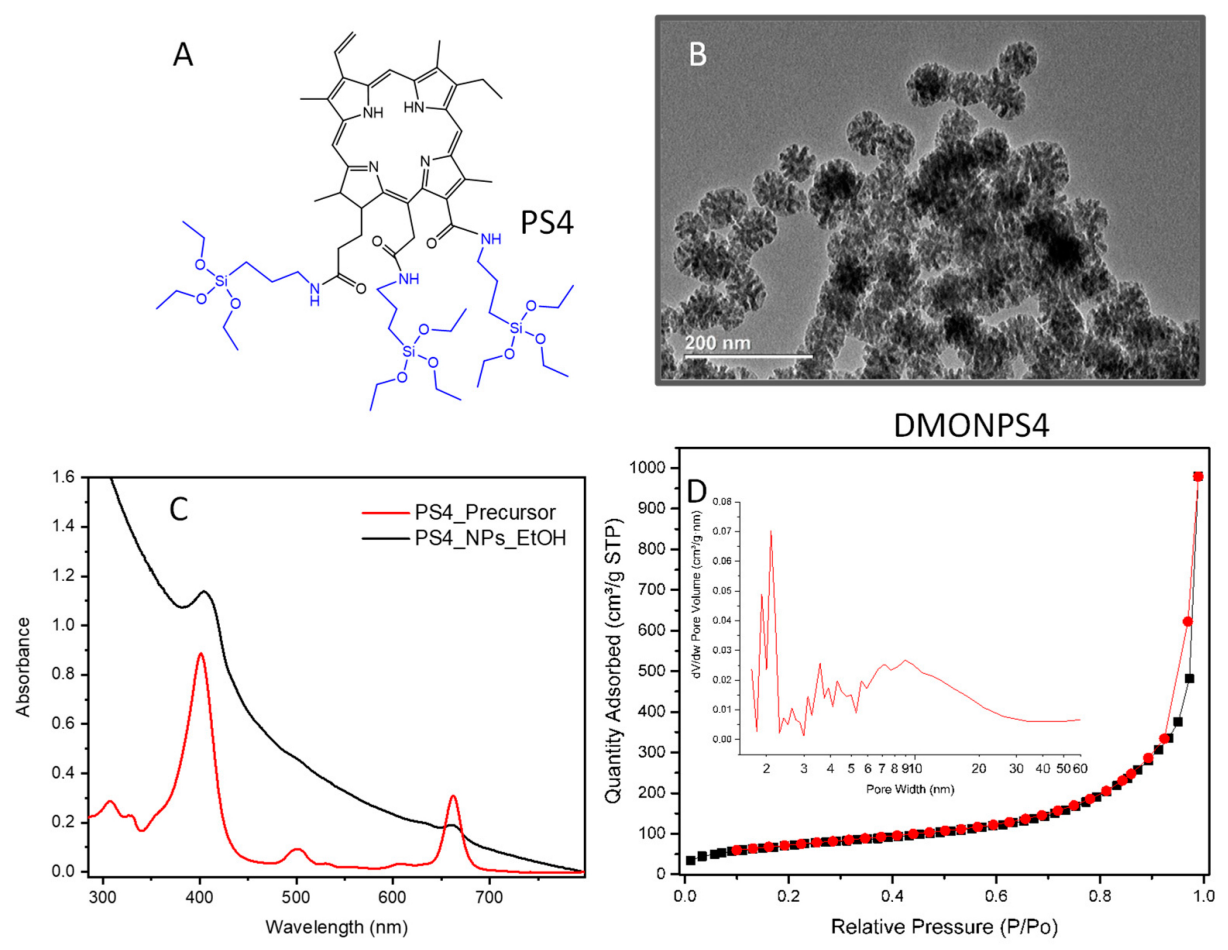
(**A**) The trisilylated chlorin e6 used for the preparation of DMONPS4. (**B**) DMONP4 as shown by TEM: the dendritic structure is visible. (**C**) UV-Vis spectra in EtOH of the silylated photosensitizer(PS4) and the corresponding DMONPS4; the photosensitizer is encapsulated inside the framework of DMONPS4, and the Soret and Q1 bands are visible. (**D**) Nitrogen adsorption–desorption at 77 K (BET). Red desorption, black adsorption. Insert BJH adsorption (dV/dw) pore volume.

**Scheme 1:**
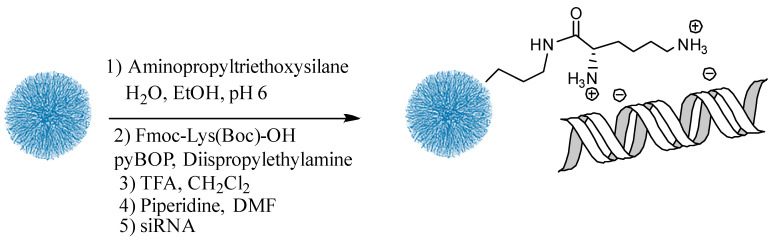
Functionalization of DMONPS1-4 with lysine amino acid (DMONPS1-4-Lys) in order to complex siRNA.

**Figure 5:**
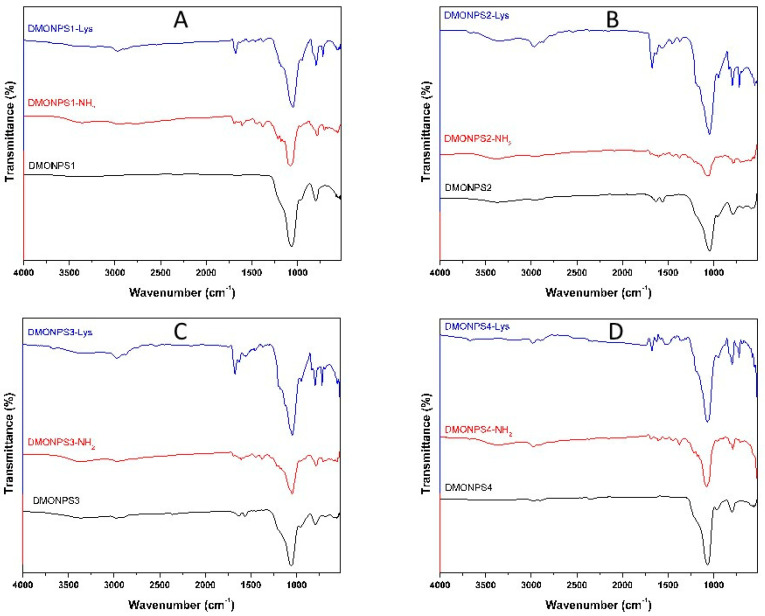
(**A**) FTIR of DMONPS1, DMONPS1-NH_2_, DMONPS1-Lys. (**B**) FTIR of DMONPS2, DMONPS2-NH_2_, DMONPS2-Lys (**C**) FTIR of DMONPS3, DMONPS3-NH_2_, DMONPS3-Lys. (**D**) FTIR of DMONPS4, DMONPS4-NH_2_, DMONPS4-Lys. All the materials showed the characteristic band at 1675 cm^−1^, corresponding to the amide I group.

DMON-PS1-4 were prepared by mixing the corresponding triethoxysilylated photosensitizers (Figure 1A, Figure 2A, Figure 3A and Figure 4A) in the presence of tetraethoxysilane, bis(triethoxysilylpropyl)tetrasulfide, following a one-pot known procedure using NaSal (sodium salicylate) and cationic surfactant CTAB as structure-directing agents and triethanolamine as a catalyst [31]. The dendritic structure and the presence of radial mesopores were clearly shown with transmission electron microscopy (TEM) (Figure 1B, Figure 2B, Figure 3B and Figure 4B) of DMONPS1-4. DLS showed well-dispersed nanoparticles from 95–100 nm and hydrodynamic diameters in agreement with TEM images. The photosensitizers were clearly incorporated inside the structure of DMONPS1-4 as shown by UV-Vis spectra. (Figure 1C, Figure 2C, Figure 3C and Figure 4C). With PS1 possessing four triethoxysilyl groups, the Q bands were visible after incorporation in DMONPS1 despite light scattering, which was not the case for DMONPS2 and DMONPS3 due to a lower amount of incorporation. Indeed, PS2 and PS3 possess only one triethoxysilyl group, which led to a less efficient anchoring of the photosensitizer inside the silica matrix. The Q1 band was present for the chlorin e6 derivative (DMONPS4) showing that the structure was not damaged during the mild sol-gel method used for the preparation of the nanoparticles. N_2_ adsorption—desorption was studied for all the DMONPS. (Figure 1D, Figure 2D, Figure 3D and Figure 4D, Table 1). All the nanoparticles showed type IV isotherms. The Brunauer−Emmett−Teller (BET) surface area and the total pore volume of DMONPS1 were 110 m^2^·g^−1^ and 0.44 cm^3^·g^−1^, respectively. The capillary condensation step occurred under the relative pressure (P/P0) of 0.8−0.9, corresponding to a large pore size of ∼24.4 nm (inset of Figure 1D). Interestingly, with DMONPS2 and DMONPS3, the BET-specific surface area increased to 356, 353 m^2^·g^−1^, with an increase in the pore volumes to 1.78, 1.65 cm^3^·g^−1^ and a decrease in the pore size to 19.5 and 18.2 nm, respectively. The structure of DMONPS4 was between DMONPS1 and DMONPS2-3 with a BET-specific surface area of 266 m^2^·g^−1^ a pore volume of 1.52 cm^3^·g^−1^ and a pore size of 22.6 nm. In order to complex siRNA, functionalization of DMONPS1-4 with lysine (Scheme 1), by amination with aminopropyltriethoxysilane (APTES) [32], coupling with protected lysine, and deprotection was performed [33]. DMONPS1-4 showed a negative zeta potential in agreement with the deprotonation of Si-OH in water. After amination, the zeta potential turned positive (Table 1), and after functionalization with lysine, high zeta potential values were observed, indicating that the functionalization was successful, also monitored by FTIR, with the appearance of the amide I band at 1675 cm^−1^ for all the materials (Figure 5).

**Figure 6:**
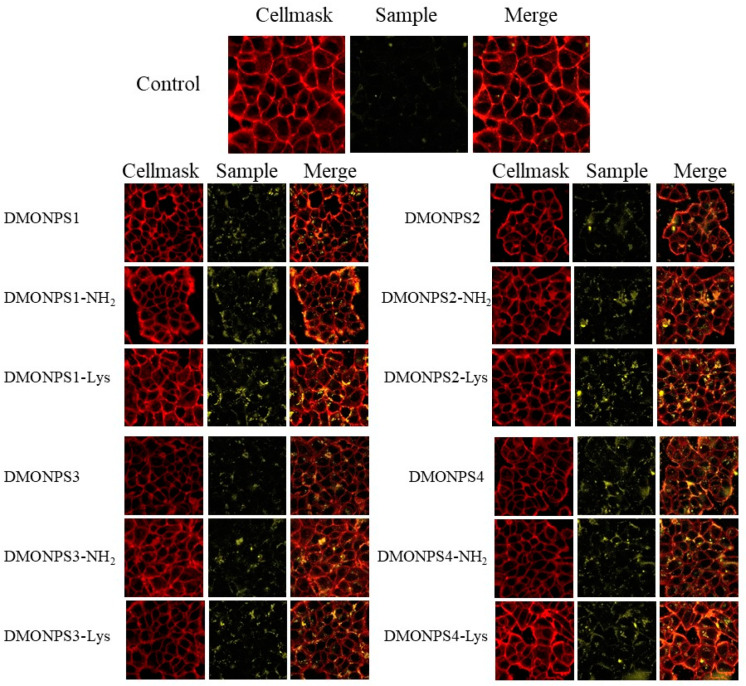
Confocal microscopy imaging of MCF-7 cancer cells incubated with DMONPS1-4, DMONPS1-4-NH_2,_ or DMONPS1-4-Lys, for 24 h. ℷ_excitation_ = 420 nm ℷ_emission_ = 630–670 nm. Membranes were stained with a cell mask 15 min before observation. ℷ_excitation_ = 561 nm ℷ_emission_ = 565–629 nm.

The nanoparticles were then incubated with cancer cells for 24 h, at a concentration of 50 µg·mL^−1^, which was adequate for imaging, and confocal microscopy was performed (Figure 6). The nanoparticles were excited at 420 nm in the Soret band of the porphyrin and chlorin. 

All the nanoparticles were endocytosed by MCF-7 cells, but DMONPS1-4-NH_2_ and DMONPS1-4-Lys with positive zeta potential were more endocytosed in the cells, in agreement with a stronger interaction of the positive charge with the cell membrane.

**Figure 7:**
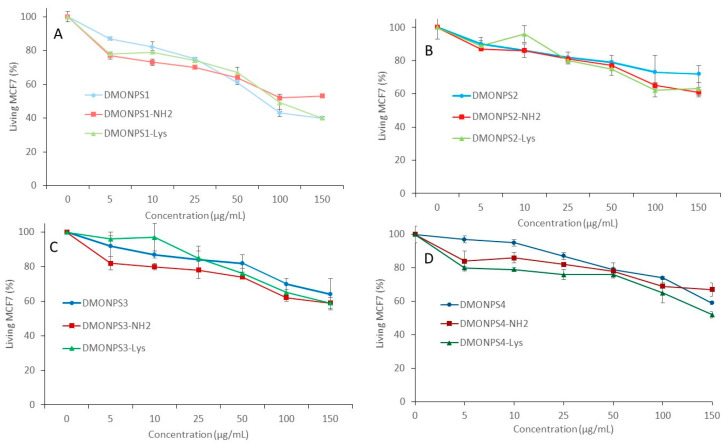
Cytotoxicity studies of MCF-7 cancer cells were performed through incubation with DMONPS1-4, DMONPS1-4-NH_2,_ or DMONPS1-4-Lys, for 72 h. The cytotoxicity was monitored with the MTT assay. Curves presented are those of DMON loaded with (**A**) PS1, (**B**) PS2, (**C**) PS3 and (**D**) PS4.

Then, cytotoxicity studies were performed (Figure 7). A total of 25 µg·mL^−1^ was the adequate concentration to carry out photodynamic therapy (PDT) experiments as the nanoparticles presented low toxicity above 75% after three days. The cells were classically irradiated [28] at 405 nm in the Soret band for 5 min or at 650 nm for 20 min in the QI band, but PDT effects were not observed. Indeed, we believe that the tetrasulfide group quenched the formation of singlet oxygen through oxidation [34]. The photosensitizers allowed for only imaging of cancer cells. 

**Figure 8:**
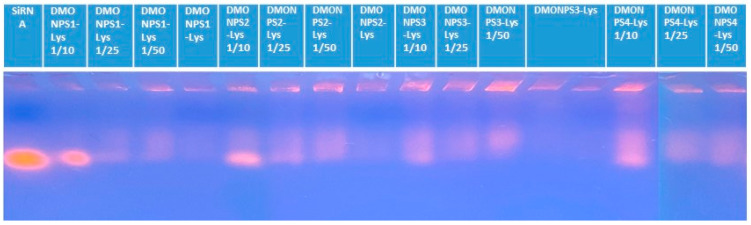
Agarose gel-retardation assay with DMONPS1-4-Lys, complexed with siRNA (negative control (A)), at different weight ratios ranging from 1/10 to 1/50. Electrophoresis was immediately performed after complex formation for 30 min at 37 °C.

Complexation of DMONPS1-4-Lys with siRNA (inhibitor apoptotic protein) IAP was then performed (Figure 8) and monitored with a gel retardation assay. Proapoptotic siRNA would inhibit the production of anti-apoptotic proteins, with the activation of the apoptotic pathway, leading to cell death. A very good complexation was noticed at concentrations of 1/25 for DMONPS1-2-lys and 1/10 for DMONPS3-4-lys.

**Figure 9:**
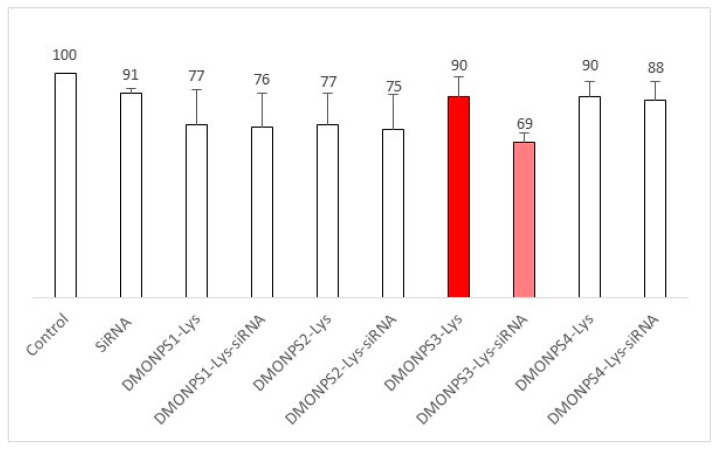
Anticancer effect of DMONPS1-4 complexed with siRNA inhibitor of apoptosis protein (IAP). MCF-7 cells were treated (or not) with free DMONPS1-4 (at 25 µg·mL^−1^) or DMONPS1-4 complexed with siRNA at a 1/15 ratio. The killing effect was monitored with the MTT assay. The red bar graphs highlighted the efficient nanoparticles for transfection.

After having determined the complexation of the siRNA/nanoparticles ratio, we then investigated the siRNA IAP delivery into MCF-7 breast cancer cells (Figure 9). We incubated the DMONPS1-4-Lys-siRNA complexes or siRNA alone with MCF-7 cancer cells for three days. MTT assay was carried out to monitor the siRNA effect (Figure 9). 

None of the nanoparticles were able to deliver the siRNA except DMONPS3-Lys. DMONPS3-Lys led to 90% MCF-7 survival whereas DMONPS3-Lys-siRNA led to 31% of cancer cell death. This result could be explained by the structure of the nanoparticles. BET analysis showed that DMONPS3 possess a high specific surface area with pore size suitable for siRNA encapsulation; furthermore, they were not aggregated with a hydrodynamic diameter suitable for the delivery of siRNA into the cytoplasm of the cells. We suggest that a high level of GSH in the cytoplasm of cancer cells allowed for the delivery of siRNA through cleavage of the tetrasulfide link present in the structure of the DMONPS3-Lys-siRNA complex. 

After that, we tested the encapsulation of the FVIII protein factor as a model protein, in DMONPS1-Lys and DMONPS3-Lys. FVIII factor is a protein that plays a crucial role in blood clotting [35]. It is produced in the liver and circulates in the blood. FVIII deficiency is the cause of hemophilia A, a genetic bleeding disorder. Hemophilia A patients require regular infusions of FVIII to prevent bleeding episodes. FVIII is administered intravenously. The half-life of FVIII in the body is approximately 8–12 h. Therefore, encapsulation of FVIII could be of high interest. Furthermore, the global charge of factor VIII is negative. This is because FVIII is a glycoprotein [36], and the carbohydrate component contains negatively charged molecules, such as sialic acid and sulfate groups, which contribute to the overall negative charge of the protein. 

We tested the encapsulation of FVIII in DMONPS1,3-Lys. DLC and DLE at 2 mg/mL of protein in PBS, and the results were quite interesting with 12–14% of loading and 35–40% of DLE (Table 2). By increasing the amount of protein in the feeding solution, higher DLC was reached with DMONPS3-Lys up to 25% of DLC.

## 3. Materials and Methods

The following chemicals, cetyltrimethylammonium bromide (CTAB), sodium salicylate (NaSal), tetraethyl orthosilicate (TEOS), bis [3-(triethoxysilyl)propyl]tetrasulfide (BTES), triethanolamine (TEA), ammonium nitrate (NH_4_NO_3_), ethanol (EtOH), dichloromethane (DCM), N,N-Diisopropylethylamine (DIPEA), isocyanatopropyltriethoxysilane, anhydrous tetrahydrofuran (THF), ethylacetate (AcOEt) and recrystallized in hexane, dimethyl sulfoxide (DMSO), (3-Aminopropyl)triethoxysilane (APTES), carbodiimide hydrochloride (EDC-HCl) and N-hydroxysuccinimide (NHS), were purchased from Sigma-Aldrich (Darmstadt, Germany). 5,10,15,20-meso-tetra(4-aminophenyl)porphyrin and 2,3,7,8,12,13,17,18-octaethylporphyrin-5-amine were purchased from TCI. 5-(4-aminophenyl)-2,3,7,8,12,18-(hexamethyl)-13,17-(diethyl)porphyrin was purchased from Porphychem (Dijon, France). Conjugated chlorin e6 (Ce6) was purchased from Frontier Scientific (Newark, NJ, USA). FVIII factor was purchased from Merck (Darmdstadt, Germany).

TEM images were recorded using a Tecnai T12 (FEI Co., Hillsboro, OR, USA) microscope operated at 120 kV. Nitrogen adsorption−desorption isotherms were acquired using a Micromeritics ASAP 2420 instrument (Micromeritics, Norcross, GA, USA). FTIR spectra were recorded using a Thermo Scientific (Waltham, MA, USA) spectrometer (Nicolet iS10) with KBr pellets. Absorption spectra were recorded using a Varian Cary 5000 spectrophotometer (Agilent, Santa Clara, CA, USA). Dynamic light scattering (DLS) and zeta potential analyses were performed using a Malvern Nano ZS instrument (Malvern Panalytical, Malvern, UK). The scattering angle was fixed at 173°. The solvent used to measure the UV-VIS spectra, DLS, and Zeta potential analyses was ethanol, with a concentration of 1 mg·mL^−1^.

### 3.1. Silylation of PS1-4

**Scheme 2:**
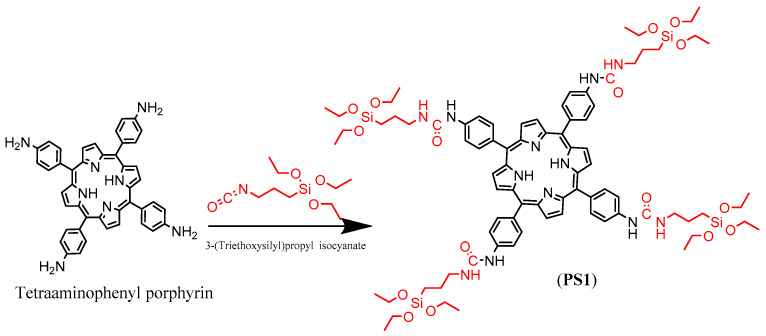
Silylation of PS1.

First, a mixture of 5,10,15,20-meso-tetra(4-aminophenyl)porphyrin (200 mg, (0.3 mmol) 1.4 × 10^−2^ mmol), DIPEA (11.2 mg), isocyanatopropyltriethoxysilane (362.8 mg (1.47 mmol)) was stirred in anhydrous THF (7 mL) under argon at 80 °C overnight (Scheme 2). After evaporation of the solvent, the POR precursor was washed with AcOEt and recrystallized in hexane. This process was repeated 4 times. Finally, the precursor was dried under vacuum [27]. 1H NMR (300 MHz, DMSO-d6) was performed to prove the silylation of the porphyrin (δ (ppm) 8.88 (s, 8H, Hβpyrrole), 8.86 (s, 4H,CO-NH-CH_2_), 8.06 (d, 3 *J* = 9.0 Hz, 8H, H3,5 aryl), 7.84 (d, 3 *J* = 9.0 Hz, 8H, H2,6 aryl), 6.40 (t, 4H, 3 *J* = 4.5 Hz, CO-NH-Ph), 3.81 (q, 3 *J* = 7.5 Hz, 24H, O-CH_2_-CH_3_), 3.2–3.16 (m; 8H NH-CH_2_-), 1.61–1.57 (m, 8H, -CH_2_-) 1.20 (t, 3 *J*= 7.5 Hz, 36H, O-CH_2_-CH_3_), 0.66 (t, 3 *J* = 9.0 Hz, 8H, CH_2_-Si), −2.8 (s, 2H, NHpyrrole) [28].

**Scheme 3:**
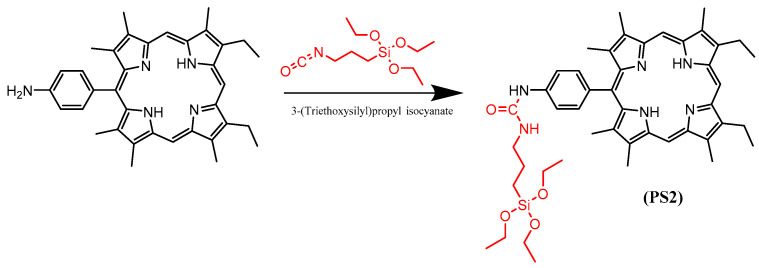
Silylation of PS2.

To a solution of 5-(4-aminophenyl)-2,3,7,8,12,18-(hexamethyl)-13,17-(diethyl)porphyrin precursor (25 mg, 0.0456 mmol) in anhydrous THF (7 mL) and DIPEA (0.015 mL, 11.2 mg), 3-isocyanatopropyltriethoxysilane (17 mg, 17 µL, 0.0684 mmol) was slowly added, and the mixture was stirred under argon at 80 °C overnight (Scheme 3). Volatiles were evaporated [28].

**Scheme 4:**
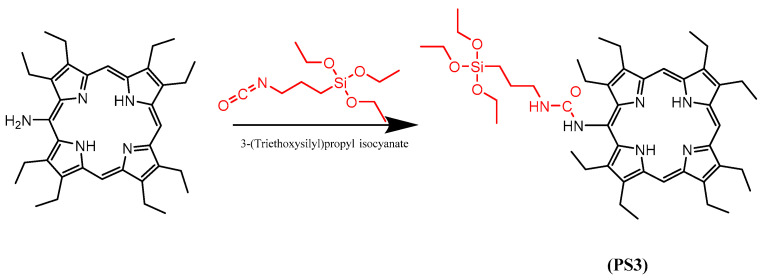
Silylation of PS3.

To a solution of 2,3,7,8,12,13,17,18-octaethylporphyrin-5-amine precursor (25 mg, 0.0455 mmol) in anhydrous THF (7 mL) and DIPEA (0.015 mL, 11.2 mg), 3-isocyanatopropyltriethoxysilane (17 mg, 17 µL, 0.0684 mmol) was slowly added, and the mixture was stirred under argon at 80 °C overnight (Scheme 4). Volatiles were evaporated [29].

**Scheme 5:**
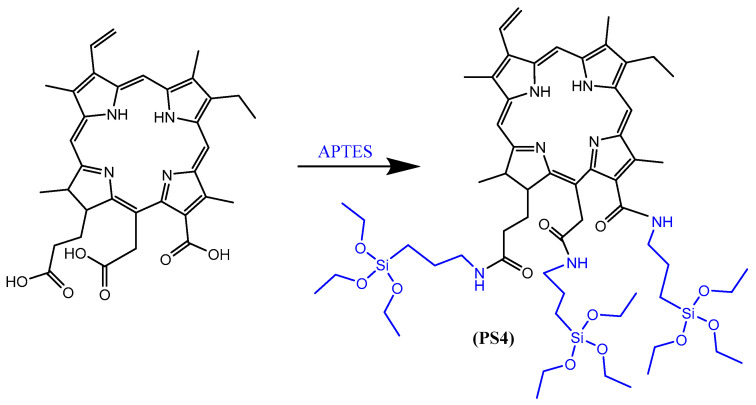
Silylation of PS4.

A total of 2.2 mg of conjugated chlorin e6 (Ce6) pre-dissolved in 0.5 mL of DMSO was mixed with 12 µL of APTES, in the presence of 6 mg of EDC-HCl and 4 mg of NHS (Scheme 5). The mixture was stirred overnight at room temperature [30].

### 3.2. Preparation of DMONPS1-4

DMONPS were synthesized via a one-pot synthesis using NaSal and cationic surfactant CTAB as structure-directing agents, TEOS and BTES as a silica source, and TEA as a catalyst. The synthesis was conducted in a 50 mL flat-bottom glass bottle with a stirring bar of 3 cm. In a typical synthesis of DMON, 0.034 g of TEA was added to 12.5 mL of water and stirred gently (∼700 rpm) at 80 °C in an oil bath under a magnetic stirrer for 0.5 h. Afterward, 190 mg of CTAB and 42 mg of NaSal were added to the above solution, which was kept stirred for another 1 h. After CTAB and NaSal were completely dissolved, a mixture of 1 mL of TEOS (Mw: 208.33, 0.0048 mmol) and 0.8 mL of BTES (Mw: 538.95, 0.00148 mmol) and PS (25 mg for PS1-3, 2.5 mg for PS4) was added to the water–CTAB–NaSal–TEA solution with vigorous stirring for 12 h. The product was recovered by centrifugation of 20,000 rpm for 5 min and washed with ethanol three times to remove the residual reactants. Finally, the yellow powder was dried in a vacuum oven at 40 °C for 6 h. Then, the collected products were extracted with HCl and methanol solution of 6 g/L NH_4_NO_3_ solution in 95% EtOH at 60 °C for 6 h three times to remove the template, followed by drying in vacuum at room temperature overnight.

### 3.3. Preparation of DMONPS1-4 -NH_2_ [32]

A total of 100 mg DMONPS was suspended with 110 mL EtOH for 10 min. Then, 2.4 mL H_2_O and 155 µL APTES were added. The pH was adjusted to 6 by the addition of HCl. The reaction was stirred at 750 rpm at room temperature for 20 h. The nanoparticles were centrifuged and washed (X3) with EtOH and then dried under vacuum.

### 3.4. Preparation of DMONPS1-4- Lys [33]

A total of 20 mg of PS NPs-NH_2_ was suspended in 2 mL of EtOH. Fmoc-Lys(Boc)-OH in the amount of 30 mg was combined with 35 mg of PyBOP and 11 µL of DIPEA The suspension was stirred for 18 h at room temperature. The nanoparticles were centrifuged, washed with EtOH, and treated with TFA/DCM solution (2 mL, 1:1) for 3 min at room temperature. After washing (X3) with EtOH, Fmoc deprotection was performed by adding a solution of Piperidine/DMF (2 mL, 1:1) for 30 min with sonication. After centrifugation for 15 min at 14,000 rpm, the supernatant was collected and used for UV-Vis spectroscopy at 290 nm wavelength.

## 4. Biological Studies

### 4.1. Cell Culture

Human breast cancer cells, MCF-7 were purchased from ATCC (Manassas, VA, USA) and cultured in Dulbecco’s Modified Eagle Medium (DMEM) supplemented with 10% FBS and 1% penicillin/streptomycin. MCF-7 was allowed to grow at 37 °C in a humidified atmosphere under 5% of CO_2_.

### 4.2. Cytotoxicity Study

MCF-7 cells were seeded into a 96-well plate, 2000 cells per well in 200 µL of culture medium, and allowed to grow for 24 h. Samples became soluble on ethanol absolute at 5 mg/mL.

Twenty hours after seeding, MCF-7 was treated with increasing concentrations from 5 to 150 µg/mL of nano. Three days after the treatment, an MTT assay was performed to determine the cell viability. Briefly, cells were incubated for 4 h with 0.5 mg/mL of MTT (3-(4,5-dimethylthiazol-2-yl)-2,5-diphenyltetrazolium bromide) in media. The MTT/media solution was then removed, and the precipitated crystals were dissolved in ethanol/DMSO (*v*/*v*). The solution absorbance was read at 540 nm in a microplate reader.

### 4.3. Confocal Fluorescent Imaging on MCF-7

Confocal fluorescent imaging was performed on MCF-7 cells seeded onto glass bottom dishes at 60,000 cells per well in 1 mL of culture medium and allowed to grow for 24 h. Then, cells were incubated for 24 h with or without nanoparticles at 50 μg·mL^−1^ for each sample. After 24 h, the incubation of cells with CellMask was performed for cell membrane staining for 15 min, and cells were washed 3 times with PBS. Fluorescence pictures were recorded on a confocal microscope (LSM 880 Zeiss Microscope, Oberkochen, Germany) under a 420 nm wavelength excitation for nanoparticle detection and a 561 nm for cell membrane imaging.

### 4.4. Gel Electrophoresis with siRNA

A total of 5 μL of a 20 μM siRNA, mixed with the appropriate amounts of nanoparticles (in order to reach the desired P+/P− ratio) in RNase-free water (final volume: 20 μL) was used to perform gel retardation assays with siRNA, and 4 μL of Blue 6X loading dye (Fisher Scientific, Hampton, NH, USA) was then added. These samples were submitted to electrophoresis performed with a 2% wt/vol agarose gel in TBE (90 mM Tris-borate/2 mM EDTA, pH 8.2) at 50 V for 1 h. The standard was a 100 bp DNA ladder (Sigma-Aldrich, Saint-Quentin-Fallavier, France). To visualize siRNA, a GelRed nucleic acid gel stain (Interchim, Montluçon, France) was used, which allowed the detection of siRNA through an ultraviolet transilluminator (Infinity Gel documentation Imaging, Vilber Lourmat, Paris, France).

### 4.5. In Vitro siRNA Delivery

MCF-7 cells were seeded in a 96-well plate at 2000 cells per well in a 100 µL culture medium. Twenty-four hours after that, the cells were incubated with or without NP or NP/siRNA at a ratio of 1/15. Three days after incubation, cells were submitted to MTT assay as described previously. The siRNA was a siRNA IAP, and the targeting sequence was inhibitor apoptotic protein (siCIAP1) 5′-CUAGGAGACAGUCCUAUUCdTdT-3′. It was purchased from Eurogentec (Serring, Belgium).

### 4.6. FVIII Encapsulation

**Figure 10:**
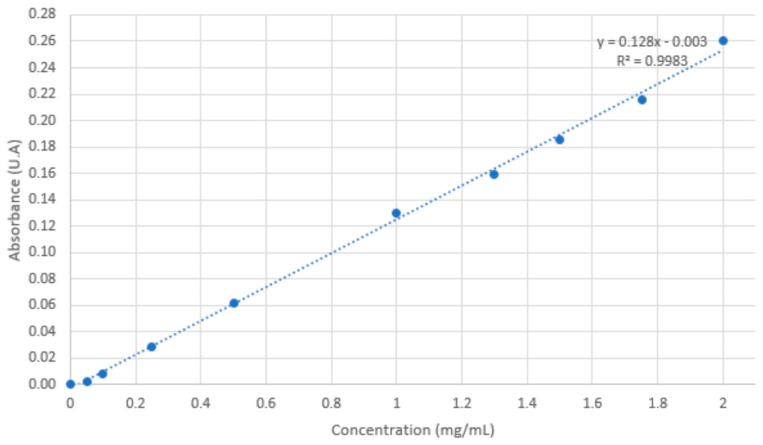
Calibration curve of FVIII at 2 mg/mL in PBS.

**Figure 11:**
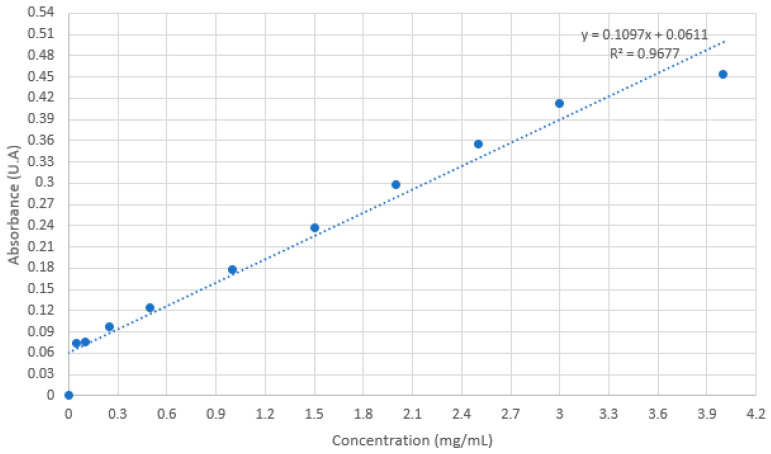
Calibration curve of FVIII at 4 mg/mL in PBS.

For the encapsulation of FVIII in DMONPSI-Lys, 5 mg of powdered nanoparticles were mixed with 5 mL of FVIII previously diluted in PBS (10 mM, pH 7.4) to obtain a final concentration of 2 mg/mL. For the encapsulation of FVIII in DMONPS3-Lys, two batches of 5 mg of powdered nanoparticles were mixed with 5 mL of FVIII previously diluted in order to have final concentrations of 2 mg/mL (Figure 10) or 4 mg/mL (Figure 11). To suspend the solution, a sonication of 5 s was carried out, and then, the solution was incubated at room temperature at 200 rpm for 24 h

$$\mathrm{DLC}\;(\%)=\frac{\mathrm{Mass of FVIII in the DMONPS}1,3-Lys}{\mathrm{Mass of DMONPS}1,3-Lys}$$

$$\mathrm{DLE}\;(\%)=\frac{\mathrm{Mass of FVIII in the DMONPS}1,3-Lys}{\mathrm{Mass of FVIII in the feeding solution}}$$

DLC and DLE were calculated after centrifugation and titration of the supernatant with UV-Vis spectra at 280 nm, using two calibration curves.

Calibration curves were determined at 2 mg/mL and 4 mg/mL of FVIII, respectively.

## 5. Conclusions

We have prepared four dendritic mesoporous organosilica nanoparticles, incorporating photosensitizers, which were then functionalized with lysine in order to first complex siRNA. Unfortunately, photodynamic therapy and photochemical internalization experiments were unsuccessful with all the porphyrin and chlorin-based materials because of the presence of the tetrasulfide bridge, which quenched singlet oxygen production. However, one formulation proved efficient for the delivery of siRNA IAP into cancer cells, without the use of PCI, as the structure of these nanoparticles was probably suitable for the delivery of siRNA into the cytoplasm of the cells. Indeed, high specific surface areas, pore volumes, and pore diameters were obtained for all the nanoparticles and all the nanoparticles complexed siRNA as shown with gel retardation assay. Furthermore, loading of FVIII factor in two formulations was efficient and high DLC and DLE were observed. Therefore, the versatility of DMON could be very useful for further nanomedicine applications and personalized medicine.

## Figures and Tables

**Table 1:** Data for DMON.

DMONPS	DLS (nm)	ZETA Potential (mV)	BET (m^2^·g^−1^)	Pore Size (nm)	Pore Volume cm^3^·g^−1^
DMONPS1	100	−8.1	110	24.4	0.44
DMONPS2	92	−8.5	356	19.5	1.78
DMONPS3	96	−10.4	353	18.2	1.65
DMONPS4	95	−7.5	265	22.6	1.52
DMONPS1-NH_2_	100	6.0	/	/	/
DMONPS2-NH_2_	99	4.8	/	/	/
DMONPS3-NH_2_	100	5.1	/	/	/
DMONPS4-NH_2_	100	6.3	/	/	/
DMONPS1-Lys	99	42.0	/	/	/
DMONPS2-lys	99	26.3	/	/	/
DMONPS3-Lys	100	26.4	/	/	/
DMONPS4-Lys	100	6.0	/	/	/

**Table 2:** Data for the encapsulation of FVIII factor in DMONPS1-Lys and DMONPS3-Lys.

DMONPS	DLC 2 mg/mL FVIII	DLE 2 mg/mL FVIII	DLC 4 mg/mL FVIII	DLE 4 mg/mL FVIII
DMONPS1-Lys	14%	40%	/	/
DMONPS3-Lys	12%	35%	25%	42%

## Sample Availability

Samples of the compounds are available from the authors.
